# The Adjunctive Role of Dynamic Systemic Inflammation-Based Biomarkers in Surgical Risk Stratification of First-Episode Primary Spontaneous Pneumothorax

**DOI:** 10.3390/diagnostics16081141

**Published:** 2026-04-11

**Authors:** Omer Topaloglu, Hasan Turut, Elvan Senturk Topaloglu, Aziz Gumus, Gokcen Sevilgen

**Affiliations:** 1 Department of Thoracic Surgery, Faculty of Medicine, Recep Tayyip Erdogan University, 53100 Rize, Turkey; omer.topaloglu@erdogan.edu.tr (O.T.); hasan.turut@erdogan.edu.tr (H.T.); gsevilgen@gmail.com (G.S.); 2 Department of Pulmonology, Faculty of Medicine, Recep Tayyip Erdogan University, 53100 Rize, Turkey; aziz.gumus@erdogan.edu.tr

**Keywords:** primary spontaneous pneumothorax, systemic inflammation, inflammation-based biomarkers, video-assisted thoracoscopic surgery, prolonged air leak, risk stratification, c-reactive protein/albumin ratio

## Abstract

**Background/Objectives:** This study examined whether dynamic systemic inflammation- and nutrition-based scores measured at baseline (T0) and during follow-up (T1: days 7–10) are associated with treatment response and surgical requirement in first-episode primary spontaneous pneumothorax (PSP). **Methods:** A total of 216 consecutive patients with first-episode PSP, treated between January 2020 and December 2024, were retrospectively analyzed. All patients initially underwent tube thoracostomy. During follow-up, 117 patients recovered with drainage therapy, whereas 99 required VATS because of a prolonged air leak. The CAR, SIII, SIRI, PIII, NLR, PLR, and PNI, measured at T0 and T1, were analyzed. Δ-values (T1–T0 differences) were evaluated, and diagnostic performance was assessed using ROC curve analysis. **Results:** At T0, inflammation- and nutrition-based indices did not differ significantly between groups. In contrast, at T1, CAR, SIII, SIRI, PIII, NLR, and PLR values were significantly higher in the VATS group than in the drainage group (all *p* < 0.05). Over time, inflammatory indices increased markedly in the VATS group, whereas changes in the drainage group remained limited. PNI decreased significantly at T1 in both groups. ROC analysis demonstrated that CAR, SIII, and NLR showed moderate discriminative performance for identifying patients who required VATS (area under the curve ≈ 0.65). **Conclusions:** Dynamic assessment of systemic inflammation-based biomarkers provides clinically relevant insight for surgical risk stratification in first-episode PSP. While baseline measurements alone are insufficient, follow-up values and temporal changes—particularly in CAR, SIII, and NLR—may reflect progression toward a surgical phenotype and could serve as adjunctive, non-directive decision-support indicators in PSP management.

## 1. Introduction

Primary spontaneous pneumothorax (PSP) is characterized by the accumulation of air in the pleural space without an identifiable underlying lung disease, and it is most commonly observed in young, tall, male smokers [1,2]. Its reported annual incidence ranges from 7 to 18/100,000 in males and 1 to 6/100,000 in females, respectively. Although most patients can be managed conservatively, progression to prolonged air leak requiring surgery remains a significant clinical challenge [3,4]. Diagnosis is primarily based on chest radiography, with computed tomography providing higher sensitivity when needed [5]. In first-episode PSP, early identification of patients who are likely to fail drainage therapy remains an important unmet clinical need [4,6].

Rupture of subpleural blebs or bullae has been described as the classic mechanism in PSP pathogenesis. However, recent evidence highlights additional mechanisms such as pleural porosity, distal airway inflammation, and disruption of the alveolo-pleural barrier integrity [2,7]. These findings suggest that PSP may not be solely a mechanical event, but a dynamic process influenced by inflammatory activity [7,8]. Emerging evidence indicates that patients with PSP may harbor underlying inflammatory and structural alterations rather than completely normal lung parenchyma [9]. Within this conceptual framework, systemic inflammatory biomarkers may serve as indirect surrogates reflecting pleural–alveolar inflammation.

Systemic inflammation-based biomarkers have gained increasing attention as indicators of disease severity and prognosis. Composite indices, such as the neutrophil-to-lymphocyte ratio (NLR), platelet-to-lymphocyte ratio (PLR), systemic immune-inflammation index (SIII), systemic inflammation response index (SIRI), pan-immune-inflammation index (PIII), and C-reactive protein/albumin ratio (CAR), have been associated with outcomes across various clinical conditions [10,11,12,13,14]. These indices integrate multiple immune pathways, providing a comprehensive representation of systemic inflammatory burden [12,13].

However, evidence suggests that the clinical relevance of these scores is more closely associated with time-dependent changes observed during follow-up [13,14,15]. Dynamic inflammatory changes may better reflect disease progression and treatment failure [14,15,16].

We therefore hypothesized that dynamic changes in systemic inflammation-based biomarkers would provide greater clinical relevance than baseline measurements alone in PSP.

Specifically for PSP, evidence on inflammation-based biomarkers and clinical course remains limited. In current clinical practice, indications for VATS are mostly determined by the duration of air leak, radiological findings, and clinical observation [4,6,17]. However, these criteria are largely reactive and may not support early risk stratification. Assessment of dynamic inflammatory changes may provide an adjunctive approach to identify patients at higher risk of conservative treatment failure [15,16,18].

The present study aimed to evaluate the clinical value of systemic inflammation- and nutrition-based scores measured at presentation and during follow-up in patients with first-episode PSP. CAR, SIII, SIRI, PIII, NLR, PLR, and PNI were analyzed in terms of baseline values and temporal changes. We further hypothesized that dynamic changes—particularly in CAR, SIII, and NLR—would be associated with progression toward persistent air leak and subsequent surgical referral, serving as adjunctive markers for clinical risk stratification.

## 2. Materials and Methods

### 2.1. Study Design and Patient Selection

This retrospective, two-stage observational study included adult patients who presented with a diagnosis of PSP between January 2020 and December 2024 and in whom a first-episode PSP was confirmed. Study data were obtained from a single center, and patients were evaluated retrospectively using the hospital information management system and archive records. Given the retrospective observational design and the absence of key clinical confounders, the study was designed to explore associations rather than establish causal or independent predictive relationships between biomarkers and surgical requirement.

Inclusion criteria for the study were as follows:

(i). Presentation to the emergency department with a diagnosis of first episode PSP;
(ii). Complete peripheral blood count and biochemical parameters available at presentation (T0);
(iii). Tube thoracostomy performed initially for all patients, and the subsequent clinical course resulted either in recovery with drainage or in VATS due to prolonged air leak (≥7 days);
(iv). In the VATS group, the follow-up blood sample at T1, obtained on day 7 prior to the surgical decision;
(v). In the drainage group, the follow-up blood sample at T1, obtained at the outpatient clinic control 7–10 days after discharge.

Exclusion criteria were as follows: secondary, traumatic, or iatrogenic pneumothorax; patients younger than 18 years; pregnancy; immunosuppression; hematologic malignancy or systemic inflammatory disease; missing laboratory data at T0 or T1; concomitant acute infection, sepsis, or any clinical condition requiring antibiotic therapy; and recurrent PSP attacks. Although PSP predominantly affects younger individuals, three patients aged 40–48 years were included after exclusion of secondary causes through thoracic computed tomography and multidisciplinary evaluation, with no radiologic or clinical evidence of underlying lung disease.

Of 193 PSP patients followed with drainage, 76 were excluded from the study because of missing follow-up data or requirement for antibiotics, and the remaining 117 patients were analyzed as the drainage group. Of 107 patients who underwent VATS for prolonged air leak, eight were excluded for similar reasons, and the remaining 99 patients with complete day-7 data were included in the VATS group. Consequently, analyses were performed on a total of 216 patients.

The study population was divided into two groups according to treatment strategy:

(1) Drainage group (*n* = 117): patients in whom air leak ceased after tube thoracostomy and who recovered with conservative therapy.

(2) VATS group (*n* = 99): patients who developed prolonged air leak despite drainage and underwent surgical intervention.

This classification was intended to evaluate the association between systemic inflammatory markers and clinical course, while acknowledging that treatment allocation was not randomized and may be influenced by unmeasured clinical factors.

### 2.2. Collection of Clinical and Laboratory Data

Laboratory values at presentation (T0) for all patients were obtained from the records of the emergency department. In patients followed with drainage, control blood samples were obtained at the planned outpatient visit during the follow-up period (T1) 7–10 days after discharge. In the VATS group, control blood values were routinely measured on day 7 during the follow-up period (T1) prior to the surgical decision. This time window was selected to reflect the early subacute phase of the systemic inflammatory response. However, T1 sampling conditions differed between groups (inpatient preoperative vs. outpatient follow-up), which may introduce measurement bias and were therefore considered in the interpretation of the results.

For complete blood count, samples were collected into EDTA-containing vacuum tubes and analyzed using an automated hematology analyzer (UniCel® DxH 800, Beckman Coulter Inc., Brea, CA, USA). Biochemical parameters (C-reactive protein (CRP) and albumin) were measured according to the hospital’s standard laboratory procedures.

The hematologic and biochemical parameters collected were as follows: CRP (mg/L), albumin (g/dL), and counts of neutrophils, lymphocytes, eosinophils, monocytes, and platelets (×10^3^/mm^3^).

### 2.3. Calculation of Indices and Scores

To evaluate the systemic inflammatory response in patients with PSP, the hematologic indices defined in the literature were calculated [19].

• Neutrophil-to-Lymphocyte Ratio (NLR) = Neutrophil/Lymphocyte.
• Eosinophil-to-Lymphocyte Ratio (ELR) = Eosinophil/Lymphocyte.
• Platelet-to-Lymphocyte Ratio (PLR) = Platelet/Lymphocyte.
• CRP/Albumin Ratio (CAR) = CRP/Albumin.
• Prognostic Nutritional Index (PNI) = [10 × Albumin] + [0.005 × Lymphocyte (cells/mm^3^)].
• Systemic Inflammation Response Index (SIRI) = (Neutrophil × Monocyte)/Lymphocyte.
• Systemic Immune-Inflammation Index (SIII) = (Neutrophil × Platelet)/Lymphocyte.
• Pan-Immune-Inflammation Index (PIII/PIV) = (Neutrophil × Platelet × Monocyte)/Lymphocyte.

All parameters were standardized according to measurement units. The T0 and T1 values were calculated, and Δ-values (T1–T0 difference) were generated to assess temporal inflammatory changes.

### 2.4. Statistical Analysis

Descriptive statistics were presented as the mean ± standard deviation or median (interquartile range, IQR) for continuous variables, and as number and percentage (%) for categorical variables. Normality of continuous variables was assessed using the Kolmogorov–Smirnov test. For between-group comparisons, Student’s *t*-test was used for normally distributed variables and the Mann–Whitney U test for non-normally distributed variables. Categorical variables were analyzed using the Chi-square test or Fisher’s exact test as appropriate. To evaluate temporal changes, T0 and T1 values of the inflammation- and nutrition-based indices were compared. The diagnostic performance of these indices for predicting the requirement for surgery (VATS) was assessed using receiver operating characteristic (ROC) curve analysis. Area under the curve (AUC) values and optimal cut-off points were determined using the Youden index. A two-tailed *p* < 0.05 was considered statistically significant for all analyses. Analyses were performed using IBM SPSS Statistics, version 26.0 (IBM Corp., Armonk, NY, USA). Because of the retrospective design, prospective sample size calculation was not feasible; therefore, post hoc power analysis was performed. Analyses based on the inflammation indices that showed the strongest association with the requirement for surgery (CAR, SIII, and NLR) showed that the statistical power was >80%. Power values obtained for the other indices were at a moderate level, and the results for these parameters should be interpreted with caution within the context of the findings. Multivariable modeling was not performed due to incomplete availability of key confounders (e.g., pneumothorax size, radiologic characteristics, and smoking status), limiting assessment of independent predictive effects. In addition, no internal validation using resampling techniques (e.g., cross-validation or bootstrapping) or external validation cohort was available; therefore, the findings should be considered exploratory and hypothesis-generating. Accordingly, the findings should be interpreted as associative rather than independently predictive.

## 3. Results

A total of 216 patients with a diagnosis of PSP were included in the study. The mean age of the patients was 30.9 ± 12.8 (range 17–48) years. Twenty patients (9.2%) were female, and 196 (90.8%) were male. The mean length of hospital stay was 7.7 ± 3.7 (range 2–28) days. Pneumothorax was most frequently observed in the right hemithorax (54.2%), followed by the left hemithorax (45.8%). All patients received tube thoracostomy as the initial treatment. During clinical follow-up, VATS was performed in 99 patients (45.8%) because of prolonged air leak, while 117 patients (54.2%) recovered with drainage therapy. Demographic, laboratory, and clinical characteristics of the patients are summarized in Table 1.

### 3.1. Comparison of Inflammation Scores Between Groups at Baseline (T0) and Follow-Up (T1)

At T0, there were no significant differences between the drainage and VATS groups with respect to systemic inflammation- and nutrition-based indices (PNI, CAR, SIRI, SIII, PIII, and NLR; all *p* > 0.05). Only the PLR-T0 value was higher in the VATS group (*p* = 0.042). In contrast, at T1, a clear separation in inflammatory markers was observed in the VATS group. The CAR, SIII, SIRI, PIII, NLR, and PLR values were significantly higher in the VATS group compared to the drainage group (all *p* < 0.05). These findings indicate that group differences became apparent during follow-up rather than at initial presentation; however, they should be interpreted cautiously due to potential confounding factors, including differences in follow-up conditions and timing of T1 measurements between groups. Between-group comparisons are presented in Table 2.

### 3.2. Analysis of Change over Time (T1–T0)

Changes in systemic inflammatory markers between T0 and T1 were evaluated according to treatment modality. In the VATS group, the CAR, SIRI, SIII, PIII, NLR, and PLR values were statistically significantly higher at T1 than at T0 (all *p* < 0.05). In the drainage group, only CAR and PLR were significantly higher at T1 than at T0, while the other inflammation indices did not change significantly. PNI decreased significantly at T1 in both groups (*p* < 0.01). Analyses of temporal changes are presented in Table 3; however, these patterns should be interpreted cautiously, as differences in clinical follow-up conditions (inpatient vs. outpatient) may have influenced the observed inflammatory trajectories.

### 3.3. ROC Analysis and Cut-Off Values

**Figure 1:**
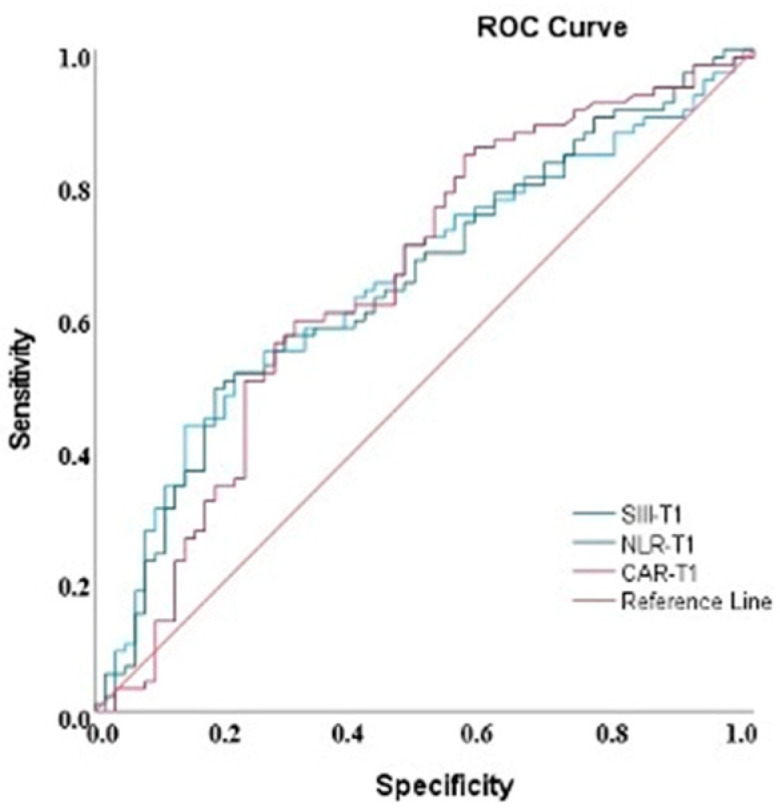
ROC curves demonstrating the diagnostic performance of CAR, SIII, and NLR indices for predicting the need for VATS.

ROC analysis demonstrated that CAR (AUC = 0.651, 95% confidence interval (CI): 0.561–0.741), SIII (AUC = 0.652, 95% CI: 0.565–0.739), and NLR (AUC = 0.651, 95% CI: 0.564–0.738) showed statistically significant but limited-to-moderate discriminative performance for identifying patients at higher risk of prolonged air leak and potential surgical referral (all *p* = 0.001). These AUC values suggest that these markers have limited standalone predictive performance and should be interpreted cautiously. ROC curves are shown in Figure 1, and diagnostic performance results are presented in Table 4.

According to the determined cut-off values, CAR ≥ 0.05 demonstrated high sensitivity (85.2%) and SIII ≥ 1500 demonstrated high specificity (90.2%). Different threshold values for NLR allowed adjustment of the sensitivity–specificity balance rather than defining a single optimal cut-off. Detailed cut-off analyses are presented in Table 5, and a translational summary of potential clinical interpretations is provided in Table 6; however, these thresholds should be considered exploratory and require external validation before routine clinical implementation.

#### Figure Findings

**Figure 2:**
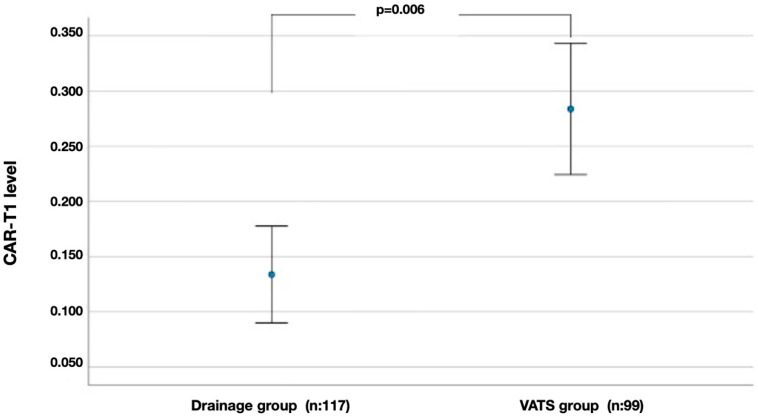
Comparison of CAR-T1 values measured at follow-up (T1) between the drainage and VATS groups. Points indicate mean values, and error bars show 95% confidence intervals.

**Figure 3:**
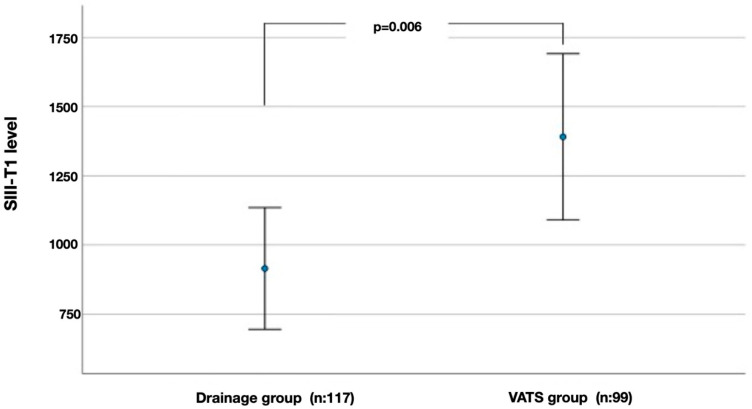
Comparison of SIII-T1 values measured at follow-up (T1) between the drainage and VATS groups. Points indicate mean values, and error bars represent 95% confidence intervals.

Error-bar plots illustrating the differences in CAR-T1 and SIII-T1 between the drainage and VATS groups showed that both parameters were markedly higher in the VATS group (Figure 2 and Figure 3).

## 4. Discussion

This study examined the role of systemic inflammation- and nutrition-based biomarkers in identifying patients at higher risk of prolonged air leak and subsequent surgical referral during follow-up in first-episode PSP. The five main findings were as follows:

(a) In patients presenting with a first episode of PSP, systemic inflammation- and nutrition-based scores measured at T0 were largely similar between patients who recovered with drainage therapy and those who subsequently required VATS because of prolonged air leak.
(b) At T1, inflammation scores were significantly higher in patients who underwent VATS compared with the drainage group. This difference was particularly pronounced for the CAR and SIII parameters.
(c) Analysis of temporal changes (T1–T0) revealed that the inflammatory response progressively increased in the VATS group. In the drainage group, inflammation remained limited and was represented mainly by CAR.
(d) The significant decrease in PNI at follow-up in both groups suggests that an acute loss of nutritional reserve develops during the early phase of PSP regardless of the subsequent clinical course.
(e) ROC analysis indicated that CAR, SIII, and NLR scores exhibited statistically significant and moderate discriminative performance for identifying patients at higher risk of persistent air leak that may be associated with a need for closer monitoring and increased clinical awareness. The identified cut-off values may provide exploratory support for clinical risk stratification; however, their clinical applicability should be interpreted cautiously given the moderate discriminative performance.

The first major finding of the present study is that, in patients with first-episode PSP, systemic inflammation- and nutrition-based indices measured at T0 do not show clear separation between patients who recover with drainage therapy and those who later require VATS due to prolonged air leak. Indeed, in our study population, composite indices such as CAR and SIII did not differ significantly between groups at T0 (CAR-T0, *p* = 0.247; SIII-T0, *p* = 0.274). This finding suggests that the systemic biomarker profile of PSP at the time of acute presentation may be insufficient, when measured at a single time point, to discriminate cases that may evolve toward a more complicated clinical course characterized by persistent air leak and the need for intensified follow-up [10,20].

This finding is biologically plausible, as the clinical relevance of systemic inflammation indices is increasingly linked to dynamic changes during disease progression rather than single time point measurements [18]. Accordingly, the lack of separation at T0 likely reflects the early and heterogeneous inflammatory response at presentation [20].

Rather than being considered a strength per se, the adoption of a dynamic assessment approach that includes not only T0 measurements but also T1 measurements addresses an expected limitation of single time point evaluation in first-episode PSP. The similarity observed at T0 should be interpreted as a reflection of the acute and heterogeneous inflammatory response at initial presentation, rather than as an indicator of methodological inadequacy. This finding underscores the limited discriminatory capacity of single baseline measurements and supports the clinical rationale for incorporating T1 and Δ-change analyses [20]. When interpreted together with the marked separation of indices such as CAR and SIII at T1 and their moderate ROC performance, these observations suggest that progression toward a more complicated clinical course, including persistent air leak, may be associated with the systemic inflammatory burden that evolves during follow-up rather than the initial inflammatory state.

The second main finding of the present study is that systemic inflammatory markers demonstrate an enhanced ability to distinguish between treatment pathways at T1. While the groups appeared similar at T0, at T1, composite indices such as CAR, SIII, SIRI, PIII, NLR, and PLR were significantly elevated in patients who later required VATS compared with those who recovered with drainage therapy. This separation was most evident for CAR-T1 and SIII-T1. These findings suggest an association between the clinical course of PSP and evolving systemic inflammatory activity, rather than implying a direct or deterministic role for these biomarkers in treatment allocation. Accordingly, the observed divergence indicates that PSP progression may be influenced not solely by baseline inflammatory burden, but by inflammation dynamics that emerge and persist during follow-up. Indeed, while the VATS group demonstrated synchronous and significant increases in most inflammatory indices from T0 to T1, the drainage group showed a more limited increase over the same interval. This pattern supports the interpretation that longitudinal changes in non-specific systemic inflammatory markers may reflect ongoing pleuro-alveolar injury and delayed healing processes, rather than serving as standalone predictors of surgical requirement. Importantly, current surgical decision-making algorithms for PSP vary across institutions and countries and are often informed by heterogeneous guidelines and gray literature rather than uniform high-level evidence. In this context, dynamic inflammatory biomarkers may provide complementary biological insight across differing clinical practice settings, rather than serving as universally directive tools.

Clinically, this separation is compatible with the hypothesis that cases progressing to prolonged air leak may have an ongoing pleuro-alveolar injury, a stress response triggered by residual air leak, and more pronounced concomitant systemic inflammatory activation. In this context, CAR’s integration of CRP’s acute-phase response with albumin as an index of nutritional reserve and indices such as SIII/PIII reflecting multiple cellular lineages simultaneously biologically rationalize the clinical separation observed at T1 [15]. In a recent study related to ours, integrated inflammation scores were also associated with adverse clinical course; these scores may strengthen holistic risk assessment as biomarkers linked to complications and unfavorable outcomes [15]. In summary, dynamic increases in indices—particularly CAR and SIII—appear to reflect progression toward a more severe PSP phenotype characterized by persistent air leak. Clinically, these findings support the use of such biomarkers for risk enrichment and prioritization of closer monitoring rather than as determinants of clinical decision-making.

The third main finding of the present study is that changes in inflammation-based indices over time (ΔT1–T0) are more informative for discriminating the requirement for surgery in PSP compared to the absolute values measured at a single time point. Our results showed that systemic inflammation indicators such as CAR, SIII, SIRI, PIII, and NLR increased significantly from their T0 values in patients who underwent VATS, whereas the inflammatory response remained more limited in patients who recovered with drainage therapy.

Given the dynamic nature of systemic inflammation, it is known that the direction and magnitude of biomarker changes during follow-up have greater potential to reflect clinical course. Indeed, progressive increases over time in composite indices such as NLR, SII/SIII, and CAR have been reported as strong indicators of adverse clinical outcomes across diverse clinical scenarios, surgical complications, and poor prognoses [10,11,14]. These studies emphasize that the inflammatory response should be evaluated as a dynamic, not a static, process.

In the specific context of PSP, the development of prolonged air leak after tube thoracostomy is associated with delayed pleural healing, persistent alveolo-pleural communication, and continuation of the local inflammatory process. The synchronous and significant increases in multiple inflammation indices observed in the VATS group in the current study present a coherent pattern, suggesting transformation of this local pathological process into a systemic manifestation [15]. In contrast, the stability of inflammation scores in the drainage group implies that in patients who achieve clinical recovery with conservative therapy, the systemic inflammatory burden can be controlled. Within this framework, Δ-analysis may contribute to clinical risk enrichment by identifying patients who may require closer surveillance during conservative management.

While single measurements at presentation are limited, evaluation of short-term increases in inflammation indices during follow-up may help identify patients in whom drainage therapy is more likely to fail.

The fourth important finding of the present study is that PNI decreased significantly at T1 in both groups of patients. However, unlike the inflammation-based indices, the decline in PNI did not discriminate between groups, suggesting that changes in nutritional reserve during the acute clinical course of PSP may reflect a generalized stress response accompanying disease burden rather than a pathway-specific phenomenon.

The prognostic value of PNI has been demonstrated in the literature, particularly in malignancies, major surgical procedures, and chronic inflammatory diseases. Low PNI values have been reported to be associated with increased risk of complications and adverse clinical outcomes [11,12]. However, PNI can decrease non-specifically during acute and short-term clinical conditions, and therefore, PNI alone should not be interpreted as a discriminatory biomarker [12]. When considered specifically in the context of PSP, tube thoracostomy, hospital admission, pain, catabolic stress, and the acute inflammatory response may cause a transient decline in serum albumin and fluctuations in lymphocyte counts. This mechanism biologically explains the similar reductions in PNI observed in both the drainage and VATS groups in the present study. Therefore, the decrease in PNI should be regarded as a systemic reflection of the acute disease process in PSP rather than as a specific indicator of the likelihood of requiring surgery [16].

The fifth key finding of our study is that the cut-off values identified by ROC analysis demonstrated statistically significant but moderate discriminative performance for identifying patients at higher risk of prolonged air leak. According to our findings, particularly the CAR, SIII, and NLR indices provided supportive risk signals for identifying patients who may require closer monitoring and enhanced clinical attention. The possible clinical interpretations of the ROC-derived cut-off values are summarized in Table 6.

The high sensitivity (85.2%) obtained for a CAR cut-off of ≥0.05 suggests that this index may be valuable for reducing the risk of delayed recognition of patients likely to develop a persistent air leak and require intensified follow-up. Conversely, the cut-off determined for SIII provided higher specificity, indicating that this index may more selectively identify patients with a pronounced systemic inflammatory phenotype who may benefit from closer clinical evaluation. Similarly, varying threshold values for NLR permit adjustment between sensitivity and specificity, supporting the use of this parameter as a flexible risk-stratification tool. These findings imply that inflammation-based indices are most appropriately applied in clinical practice as adjunctive tools for risk enrichment and follow-up prioritization rather than standalone diagnostic or determinants of clinical decision-making. The literature generally reports AUC values for many inflammation indices in the range of 0.60–0.75; such levels are considered adequate in the clinical context for early risk stratification and for identifying patients who warrant closer surveillance [10,14,21]. These findings should be interpreted with caution, as AUC values in this range indicate limited-to-moderate predictive performance and do not support strong standalone clinical utility.

In addition to the composite inflammation indices evaluated in the present study, emerging biomarkers such as butyrylcholinesterase (BChE) have recently gained attention as indicators of systemic inflammatory burden and postoperative risk. Previous observational and prospective studies have demonstrated that decreased perioperative BChE levels are associated with higher surgical site infection rates, increased complication severity, and adverse postoperative outcomes, suggesting that BChE may represent a complementary, non-specific inflammatory marker worthy of further investigation alongside established indices in future PSP-focused studies [22,23].

In routine PSP management, indications for VATS are most often determined reactively on the basis of air leak duration, radiological findings, and clinical course. These criteria frequently result in the decision being made after the need for surgery has already arisen. The CAR, SIII, and NLR cut-offs identified in our study may offer clinicians additional objective data to contribute to risk stratification, particularly in identifying patients who may benefit from closer monitoring or closer clinical follow-up. Importantly, these biomarkers are not intended to direct clinical decision-making but to complement established clinical criteria by highlighting patients who may benefit from intensified surveillance during conservative management.

Future studies should focus on integrating clinical, radiological, and laboratory variables into multivariable predictive models. In this context, advanced approaches such as LASSO regression and machine learning–based models may improve predictive performance. Previous studies have demonstrated that such models provide better discrimination and calibration compared to single-parameter approaches [24]. Therefore, prospective, multicenter studies with internal and external validation are warranted.

### Limitations

This study has several limitations. First, its retrospective design necessitates interpreting the observed associations as correlational rather than causal. Retrospective data collection from patient records may have permitted unmeasured inter-center or practice variability in measurements and clinical decision processes. Second, the study population focused on a single clinical scenario (first-episode PSP); secondary pneumothoraces and recurrent cases were excluded. This limitation restricts the generalizability of the results across the entire pneumothorax spectrum. However, restricting inclusion to first-episode PSP patients increased cohort homogeneity and thereby facilitated a clearer assessment of the relationship between inflammation-based biomarkers and the requirement for surgery. Third, inflammation- and nutrition-based scores are non-specific biomarkers and may be affected by factors outside of pneumothorax, such as acute stress, pain, length of hospitalization, and subclinical infections. Although patients with concurrent infection, antibiotic use, or missing data were excluded, it is not possible to eliminate all potential confounders that might influence these parameters. Fourth, the cut-off values derived from ROC analysis are specific to our study population and may vary in different centers, patient profiles, or prospective cohorts. Therefore, the identified thresholds should be interpreted not as surgical indication criteria but as exploratory markers for risk enrichment that require external validation and should be interpreted in conjunction with clinical, radiological, and patient-specific factors. Finally, although we evaluated temporal changes in inflammation indices, the lack of more frequent serial measurements or inclusion of additional biomarkers (for example, cytokine profiles) may have limited our ability to delineate the biological depth of the inflammatory process. This limitation highlights an important avenue for future prospective, multicenter research. In addition, no internal validation using resampling techniques (e.g., cross-validation or bootstrapping) or external validation cohort was available, which limits the generalizability of the findings.

## 5. Conclusions

This study demonstrates that dynamic assessment of systemic inflammation- and nutrition-based scores provides clinically relevant information for predicting surgical requirement during follow-up in patients with first-episode PSP. While inflammation indices measured at initial presentation were not discriminatory, the divergence of inflammation-based scores at T1—particularly CAR, SIII, and NLR—suggests that patients progressing to surgery exhibit a distinct, time-dependent systemic inflammatory phenotype.

The cut-off values identified by ROC analysis indicate that these biomarkers should be considered as adjunctive tools for early risk stratification and closer clinical surveillance rather than as standalone criteria for surgical decision-making. In contrast, the similar decline in PNI observed in both groups supports the interpretation that nutritional reserve reduction reflects an acute systemic response rather than a surgery-specific signal.

Overall, evaluation of temporal changes in inflammation-based biomarkers may aid in the early identification of patients at risk of failure of conservative management. Further prospective, multicenter studies are warranted to validate these findings and to define their role in clinical algorithms.

## Figures and Tables

**Table 1:** Demographic and clinical characteristics of patients with first-episode primary spontaneous pneumothorax.

Age (years, mean ± SD) (range)	**30.9 ± 12.88 (17–48)**
Sex	
Female (%)	20 (9.2%)
Male (%)	196 (90.8%)
Length of hospital stay (days, mean ± SD) (range)	7.7 ± 3.7 (2–28)
Pneumothorax location	
Right hemithorax	117 (54.2%)
Left hemithorax	99 (45.8%)
Treatment applied	
Drainage	117 (54.2%)
VATS	99 (45.8%)

Data are presented as number (percentage), mean ± standard deviation, and median (IQR 25–75%).

**Table 2:** Comparison of systemic inflammation- and nutrition-based indices between drainage and VATS groups at baseline (T0) and follow-up (T1).

	Drainage (*n*: 117)	VATS (*n*: 99)	*p*-Value
PNI-T0	45 (42–48)	45 (42–46)	0.172
PNI-T1	44 (39–47)	42 (40–44)	0.119
CAR-T0	0.06 (0.02–0.18)	0.08(0.02–0.16)	0.247
CAR-T1	0.09 (0.02–0.19)	0.19 (0.09–0.45)	**0.001**
SIRI-T0	1.51 (0.99–2.32)	1.68 (0.97–2.89)	0.324
SIRI-T1	1.18 (0.86–1.96)	2.16 (1.14–4.21)	**0.034**
SIII-T0	630 (450–827)	662 (441–1045)	0.274
SIII-T1	555 (381–878)	906(497–1682)	**0.006**
PIII-T0	348 (220–547)	380 (224–720)	0.426
PIII-T1	320 (198–476)	520 (278–1018)	**0.019**
NLR-T0	2.66 (1.81–3.79)	2.87 (2.01–4.50)	0.192
NLR-T1	2.36 (1.54–3.35)	3.73 (2.15–7.50)	**0.019**
PLR-T0	103 (83–125)	120 (82–159	**0.042**
PLR-T1	117 (92–154)	142 (100–192)	**0.012**

Data are presented as mean ± standard deviation, median (IQR 25–75), and count where applicable. Bold values indicate statistically significant differences (*p* < 0.05).

**Table 3:** Changes in systemic inflammation- and nutrition-based indices between T0 and T1 in drainage and VATS groups.

	Drainage (*n*: 117)			VATS (*n*: 99)		
	**T0**	**T1**	***p*** **-Value**	**T0**	**T1**	***p*** **-Value**
PNI	45 (42–48)	44 (39–47)	**0.002**	45 (42–46)	42 (40–44)	**<0.001**
CAR	0.06 (0.02–0.18)	0.09 (0.02–0.19)	**0.004**	0.08 (0.02–0.16)	0.19 (0.09–0.45)	**0.001**
SIRI	1.51 (0.99–2.32)	1.18 (0.86–1.96)	0.277	1.68 (0.97–2.89)	2.16 (1.14–4.21)	**0.018**
SIII	630 (450–827)	555 (381–878)	0.462	662 (441–1045)	906(497–1682)	**0.005**
PIII	348 (220–547)	320 (198–476)	0.565	380 (224–720)	520 (278–1018)	**0.031**
NLR	2.66 (1.81–3.79)	2.36 (1.54–3.35)	0.151	2.87 (2.01–4.50)	3.73 (2.15–7.50)	**0.004**
PLR	103 (83–125)	117 (92–154)	**0.004**	120 (82–159)	142 (100–192)	**0.010**

Wilcoxon rank test.Bold values indicate statistically significant differences (*p* < 0.05).

**Table 4:** ROC analysis results for CAR, SIII, and NLR to determine their efficiency in predicting the need for VATS.

	Area	Standard Error	95% Confidence Interval	*p*-Value
CAR	0.651	0.046	0.561–0.741	0.001
SIII	0.652	0.044	0.565–0.739	0.001
NLR	0.651	0.044	0.564–0.738	0.001

**Table 5:** Cut-off values and diagnostic performance measures for systemic inflammation-based indices to determine their efficiency in predicting the requirement for VATS.

Cut-Off Values	Sensitivity	Specificity	PPD	NPD
CAR ≥ 0.05	85.2%	40.1%	65.8%	67.5%
CAR ≥ 0.20	44.7%	77.3%	73.7%	52.6%
SIII ≥ 800	59.1%	65.7%	59.8%	54.5%
SIII ≥ 1500	29.6%	90.2%	74.4%	75.1%
NLR ≥ 1.5	88.8%	15.7%	50.3%	59.3%
NLR ≥ 5	35.7%	80.4%	63.6%	56.6%

**Table 6:** Translational summary of ROC-derived cut-offs for CAR, SIII, and NLR, indicating risk enrichment for subsequent VATS due to prolonged air leak in first-episode PSP.

Index	Cut-Off Value	Diagnostic Feature	Clinical Interpretation	Possible Clinical Approach
CAR	≥0.05	High sensitivity	Early detection of patients at higher risk for persistent air leak	Close clinical monitoring; assess inflammatory trend and clinical course
CAR	≥0.20	High specificity	Higher likelihood of persistent air leak and failure of conservative management	Higher likelihood of persistent air leak and failure of conservative management
SIII	≥1500	High specificity	Pronounced systemic inflammatory phenotype during follow-up	Escalate surveillance; integrate biomarkers with imaging and clinical findings
NLR	≥1.5	High sensitivity	Early inflammatory risk enrichment signal	Frequent follow-up; monitor dynamic changes in inflammatory markers
NLR	≥5.0	High specificity	Advanced inflammatory response	Escalate monitoring; integrate biomarkers with clinical course, and arrange early surgical consultation if air leak persists

## Data Availability

All data generated or analyzed during this study are included in this article. The data will be available upon reasonable request (contact persons: omer.topaloglu@erdogan.edu.tr).
